# A preliminary study of linear accelerator-based spatially fractionated radiotherapy

**DOI:** 10.3389/fonc.2024.1495216

**Published:** 2025-01-14

**Authors:** Young Kyu Lee, Yunji Seol, Byeong Jin Kim, Kyu Hye Choi, Ji Hyun Hong, Chan-beom Park, Sun Hwa Kim, Hyeong Wook Park, Wonjoong Cheon, Young Nam Kang, Byung‑Ock Choi

**Affiliations:** ^1^ Department of Radiation Oncology, Seoul St. Mary’s Hospital, College of Medicine, The Catholic University of Korea, Seoul, Republic of Korea; ^2^ Department of Biomedicine & Health Sciences, College of Medicine, The Catholic University of Korea, Seoul, Republic of Korea; ^3^ Department of Medical Physics, Kyonggi University, Suwon, Republic of Korea

**Keywords:** spatially fractionated radiation therapy (SFRT), lattice radiation therapy (LRT), multi-leaf collimator (MLC), peak-to-valley dose ratio (PVDR), monitor unit (MU) analysis

## Abstract

**Purpose:**

This study aimed to provide quantitative information for implementing Lattice radiotherapy (LRT) using a medical linear accelerator equipped with the Millennium 120 multi-leaf collimator (MLC). The research systematically evaluated the impact of varying vertex diameters and separations on dose distribution, peak-to-valley dose ratio (PVDR), and normal tissue dose.

**Methods:**

A cylindrical Virtual Water™ phantom was used to create LRT treatments using the Eclipse version 16.0 treatment planning system (Varian, Palo Alto, USA). The plans were optimized employing a 3 × 3 × 3 lattice structure with vertex diameters ranging from 0.5 to 2.0 cm and separations from 1.0 to 5.0 cm. The prescribed dose was 20.0 Gy to 50% of the vertex volume in a single fraction. Peak-to-valley dose ratio (PVDR) was calculated along three orthogonal axes, and normal tissue dose and monitor units (MU) were analyzed. Additionally, the modulation complexity score (MCS) was calculated for each plan to quantitatively assess treatment plan complexity.

**Results:**

The PVDR analysis demonstrated heterogeneous dose distribution, with optimal values below 30% in all directions for 5.0 cm separation. PVDR in the superior-inferior direction was consistently lower than in other directions. Normal tissue dose analysis revealed increasing mean dose with larger diameters and separations, while the volume receiving high doses decreased. MU analysis showed significant contributions from collimator angles of 315.0° and 45.0°. MCS values ranged from 0.02 to 0.17 for 0.5 cm vertex diameter and 0.08 to 0.20 for larger diameters (1.0-2.0 cm) across different separations, respectively.

**Conclusions:**

This study demonstrates the technical feasibility of implementing LRT using a medical linear accelerator with Millennium 120 MLC. The findings provide insights into optimizing LRT treatment plans, offering a comprehensive quantitative reference for achieving desired dose heterogeneity while maintaining normal tissue protection.

## 1 Introduction

Radiation Therapy is widely used as a primary technique for cancer treatment, and technological advancements have enhanced its efficacy and applicability. Spatially fractionated radiotherapy (SFRT), an emerging technique, subdivides the target tumor volume into multiple segments, with some segments receiving high-dose radiation, called ‘vertex’ (peaks), while maintaining lower doses in surrounding regions (valleys) (1). This study seeks to optimize therapeutic outcomes by delivering escalated doses to tumorous tissue while mitigating radiation exposure to adjacent healthy structures (2–4).

SFRT offers various advantages in tumor treatment. Compared to conventional whole-field radiotherapy, SFRT delivers lower doses to normal tissue, thereby reducing radiation-induced side effects. The safety and efficacy of SFRT have been demonstrated through numerous clinical studies as well as radiobiological and immunological research (5–9). Recent studies have reported that SFRT can enhance the efficiency of cancer treatment by leveraging fundamental radiobiological mechanisms such as the bystander effect, vascular damage, and activation of anti-cancer immune responses (10, 11). Specifically, the clinical effectiveness of SFRT has been observed when the peak-to-valley dose ratio (PVDR) of high-dose peaks to low-dose valleys is maintained between 20% and 30% (12–14).

SFRT was initially implemented and studied using specialized equipment in the form of GRID blocks. However, this approach presented various limitations, including difficulties in accurately calculating and measuring beam distribution, technical complexities, and challenges in maintaining appropriate dose levels in both tumor and surrounding normal tissues (15). Advances in radiotherapy technologies, such as volumetric modulated arc therapy (VMAT) and the introduction of multi-leaf collimators (MLC), have made it possible to implement SFRT in a new form called lattice radiation therapy (LRT) (16). MLC allow for the shaping of radiation beams according to the specific anatomy of the tissue and tumor, offering greater flexibility and precision in delivering radiation to the tumor compared to traditional GRID forms. Studies suggests that these technological improvements in SFRT can enhance therapeutic outcomes while reducing normal tissue toxicity (17–21).

Despite advancements in radiotherapy equipment, the effective clinical application of LRT remains challenging. A critical factor in its successful implementation is the quantitative understanding of achievable PVDR for specific equipment, as PVDR is heavily dependent on the size and spacing of high-dose vertices. This knowledge is crucial for optimizing treatment plans and ensuring therapeutic efficacy.

While most current studies have focused on clinical applications, systematic investigations of PVDR achievability across various radiotherapy machines are scarce. This technical research will provide valuable insights for optimizing LRT implementation in clinical settings. To address this, our study focuses on implementing LRT using a TrueBeam medical linear accelerator (Varian Medical Systems, Palo Alto, USA) quipped with Millennium 120 MLC. We aim to quantitatively analyze the relationship between PVDR and the diameter and separation of vertices in this specific setup. By systematically varying these parameters and calculating the PVDR, we intend to establish a comprehensive understanding of the feasible PVDR range for this equipment. This analysis will provide valuable insights for clinicians and medical physicists, enabling more informed decision-making in treatment planning and potentially expanding the practical applications of LRT in cancer treatment.

## 2 Materials and methods

### 2.1 Phantom selection and 3D lattice design

**Figure 1:**
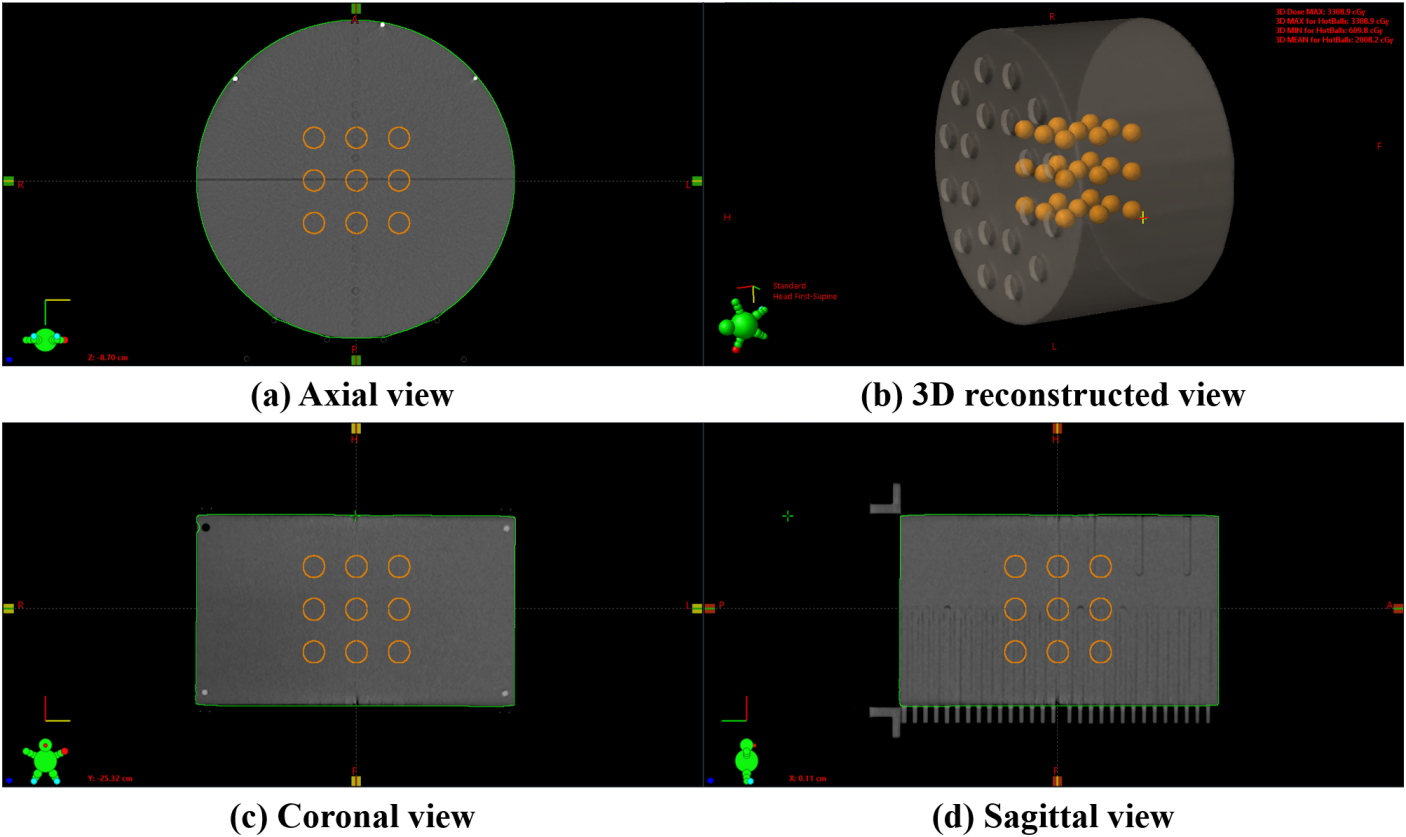
An example of a 3D lattice structure in the Virtual Water™ phantom: **(A)** axial view showing the cross-sectional arrangement of vertices, **(B)** a 3D reconstructed view demonstrating the overall spatial distribution, **(C)** coronal view showing the superior-inferior vertex distribution, and **(D)** sagittal view illustrating the anterior-posterior vertex arrangement. The internal orange spheres represent the vertices.

For the implementation and PVDR analysis of LRT using medical linear accelerators, as shown in **Figure 1**, we used a cylindrical Virtual Water™ phantom (Gammex RMI, Middleton, WI), which demonstrates the 3D lattice structure from (a) axial view, (b) 3D reconstructed view, (c) sagittal view, and (d) coronal view. This phantom is equivalent to water in radiation absorption and scattering properties. CT images of the phantom were acquired using the SOMATOM go.Open Pro CT scanner (Siemens Healthineers, Germany) with a slice thickness of 1.0 mm.

Using in-house software, we created a 3 × 3 × 3 lattice structure within the phantom, consisting of twenty-seven high-dose vertices. The diameters of these vertices ranged from 0.5 cm to 2.0 cm in 0.5 cm increments. The separation, defined as the edge-to-edge distance between adjacent vertices, varied from 1.0 cm to 5.0 cm in 1.0 cm increments. This resulted in 20 distinct series, each with a unique combination of vertex diameter and separation. Radiation therapy plans were established for each of these series.

### 2.2 Radiation treatment planning

Radiation treatment plans were optimized for delivery on a TrueBeam equipped with a Millennium 120 MLC. The Eclipse version 16.0 treatment planning system (Varian Medical Systems, Palo Alto, USA) was employed for radiation treatment planning. The prescribed dose was set to deliver 20.0 Gy to 50% of the vertex volume in a single fraction. All radiation treatment plans utilized 6 MV flattening filter-free (FFF) beams with a dose rate of 1400 MU/min. Four complete arcs were used for treatment delivery, with each arc having a unique collimator angle (0.0°, 45.0°, 90.0°, or 315.0°). All radiation treatment plans used in this study were coplanar plans with a table angle of 0°. The optimization was performed using the photon optimizer (PO) algorithm, and the final dose was calculated using the anisotropic analytical algorithm (AAA) with a 1.0 mm dose calculation grid size.

**Figure 2:**
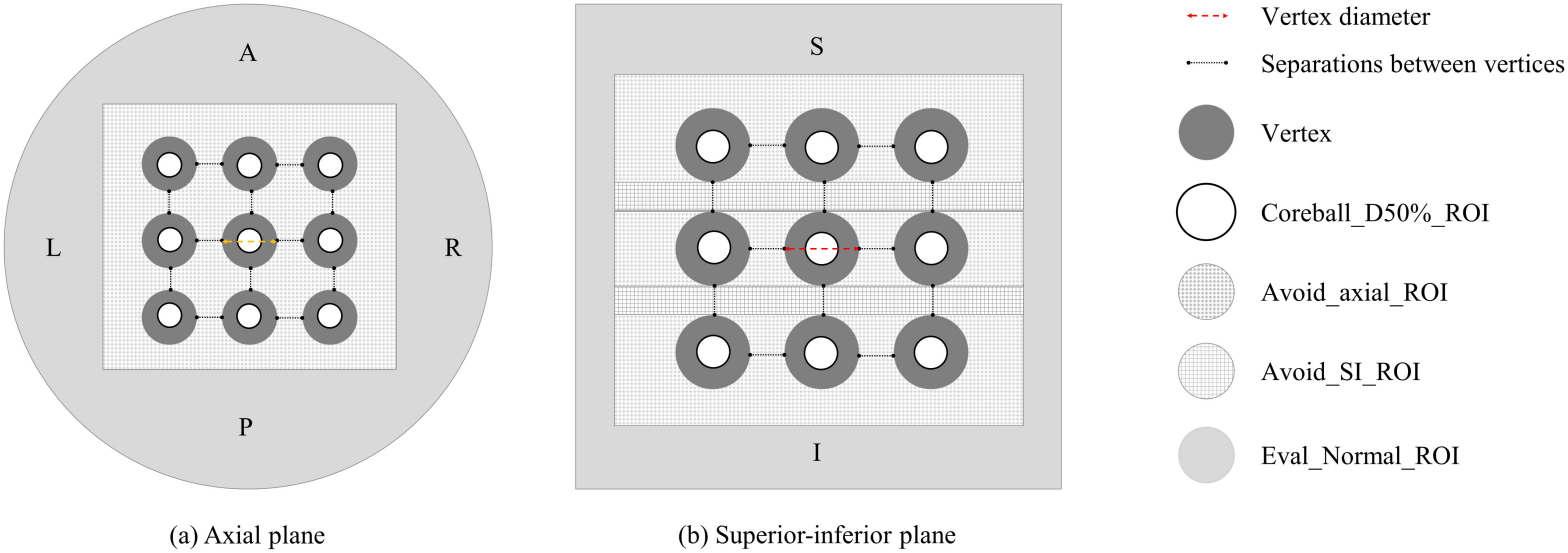
Vertices and regions of interest (ROI) configuration: **(A)** axial plane and **(B)** superior-inferior plane views showing the arrangement of vertices and various planning ROIs.

As shown in **Figure 2**, various regions of interest (ROIs) were established to achieve the planning objectives. The coreball_D50%_ROI was defined to incorporate a spherical region with its diameter equal to half that of the vertex, positioned at the center of each vertex to ensure maximum dose delivery at the center point. A total of twenty-seven coreball_D50%_ROIs were generated independently, enabling individual dose distribution control for each vertex. In the treatment planning optimization process, the minimum dose of coreball_D50%_ROI was set to the prescription dose of 20.0 Gy, and the maximum dose was set to 22.0 Gy, which is 110% of the prescription dose. The optimization priority was adjusted to ensure that 50% of the vertex volume receives 50% of the prescription dose. Through independent control of each vertex, we could ensure that the minimum dose met the prescription dose while maintaining dose uniformity between vertices.

Avoid_axial and avoid_SI are two ROIs created to minimize the dose between the vertices. These ROIs are dynamically configured based on the diameter and separation of the vertices. Each ROI serves to reduce radiation dose in the surrounding area, excluding the high-dose region of the vertices themselves. The avoid_axial ROI focuses on dose reduction in the axial plane, particularly in the X and Y-axis directions, while the avoid_SI ROI emphasizes dose reduction in the superior-inferior (SI) direction along the Z-axis. Both avoid_ROIs are formed as rectangular prisms extending 1.0 cm from the outermost vertex ends, ensuring that their dimensions adapt according to variations in the diameter or separation of the vertices.

Eval_Normal_ROI was created to assess the dose to normal tissue. Eval_Normal_ROI is defined as the entire phantom volume excluding the avoid_ROIs. This configuration allows for the evaluation of dose to surrounding normal tissue, assuming the vertices are located within tumors.

**Figure 2** illustrates the configuration of vertices and ROIs in axial and SI planes for the radiation treatment plan. It shows the arrangement of high-dose vertices, Coreball_D50%_ROI, and avoidance structures (Avoid_axial_ROI and Avoid_SI_ROI). This procedure facilitates the optimization of dose delivery to the vertices while enabling the evaluation of dose distribution in surrounding normal tissue.

### 2.3 Calculate the peak-valley dose ratio

**Figure 3:**
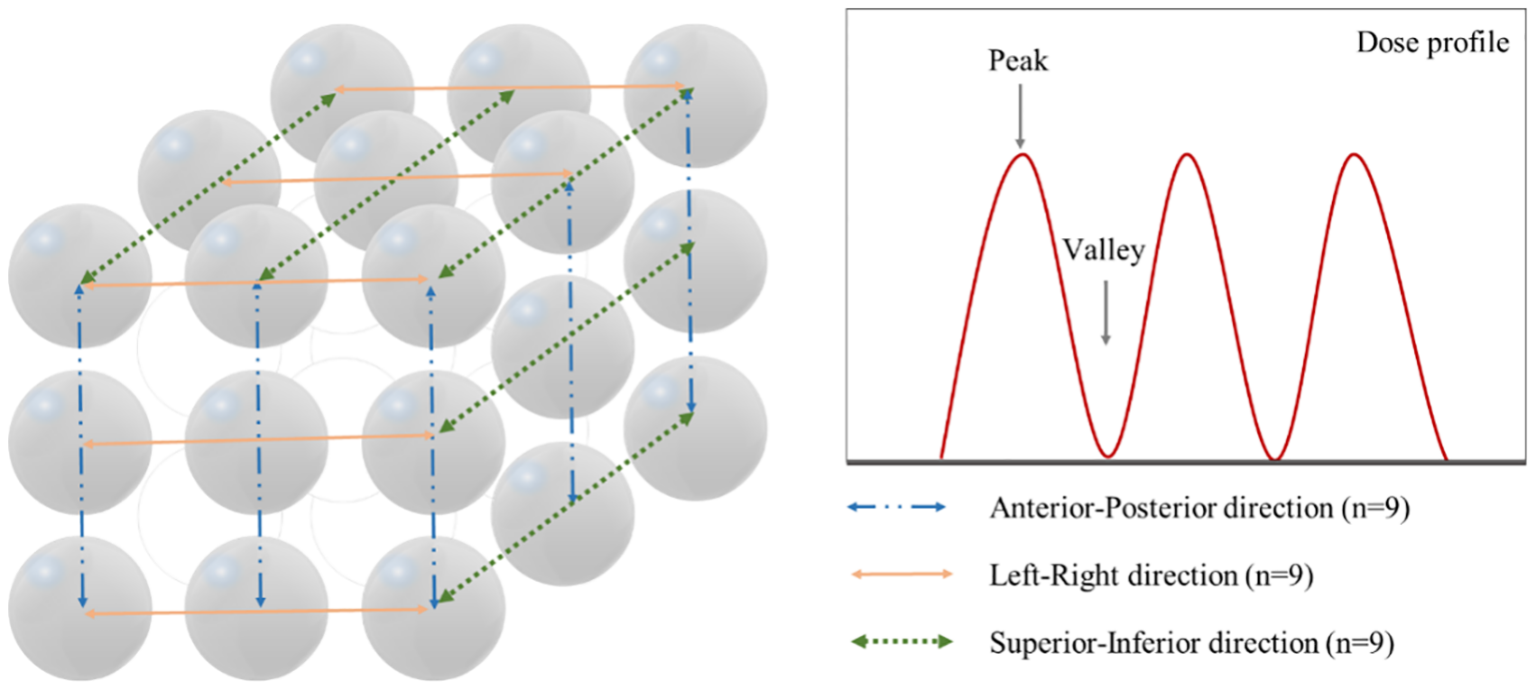
Method for acquiring dose profiles in three orthogonal directions and identification of peak and valley dose locations. Left panel shows the measurement paths along the anterior-posterior (n=9, blue dash-dot line), left-right (n=9, orange solid line), and superior-inferior (n=9, green dotted line) directions. Right panel demonstrates a representative dose profile with peak and valley points.


PVDR values are used as a significant metric for evaluating the dose distribution characteristics and treatment effectiveness of SFRT. To calculate the PVDR, as shown in **Figure 3**, a total of 27 dose profiles were obtained in each plan from the anterior-posterior (AP), left-right (LR), and SI directions, demonstrating the methodology for acquiring dose profiles and identifying peak and valley dose locations. The maximum dose points at each vertex were determined dosimetrically to obtain the dose profiles. For each direction, a single line connecting the maximum dose points of three vertices was drawn, and dose profiles were obtained along these lines. Subsequently, peak dose and valley dose values were identified from the dose profiles, and the ratio between these two values was calculated. The PVDR is defined by the following formula:

(1) $$\text{PVDR}=\frac{D_{valley}}{D_{peak}}\;\times \;100,$$

Where $D_{peak}$ represents the maximum dose (peak dose) measured in the dose profile, and $D_{valley}$ represents the minimum dose (valley dose) measured in the dose profile.

The mean and standard deviation of the PVDR obtained for each direction were calculated.

### 2.4 Normal tissue dose, volume, and plan complexity analysis by diameter and separation

Quantitative analyses were performed to evaluate the dosimetric performance of LRT plans. The mean dose within Eval_Normal_ROI was measured, and the volume intersecting the 50% prescription dose region (intersecting volume) was extracted to analyze the high dose delivered to normal tissues.

To evaluate the efficiency of the treatment plans, four arcs with different collimator angles were utilized. The total monitor units (MU) for each plan were analyzed to assess the characteristics of each plan. Additionally, to quantify the contribution of each arc within an individual treatment plan, the MU ratio of each arc relative to the total MU was calculated. The modulation complexity score (MCS), was calculated to evaluate plan complexity, which considers leaf sequence variability and aperture area variability, expressed as a value between 0 and 1 (with 0 indicating highest complexity and 1 indicating lowest complexity) (22).

A comprehensive analysis of dose distribution and normal tissue protection was conducted for each LRT plan. This analysis provides valuable insights for optimizing and evaluating LRT treatment plans, offering a quantitative foundation for creating clinically acceptable LRT plans.

## 3 Results

### 3.1 Calculate the peak-valley dose ratio

**Figure 4:**
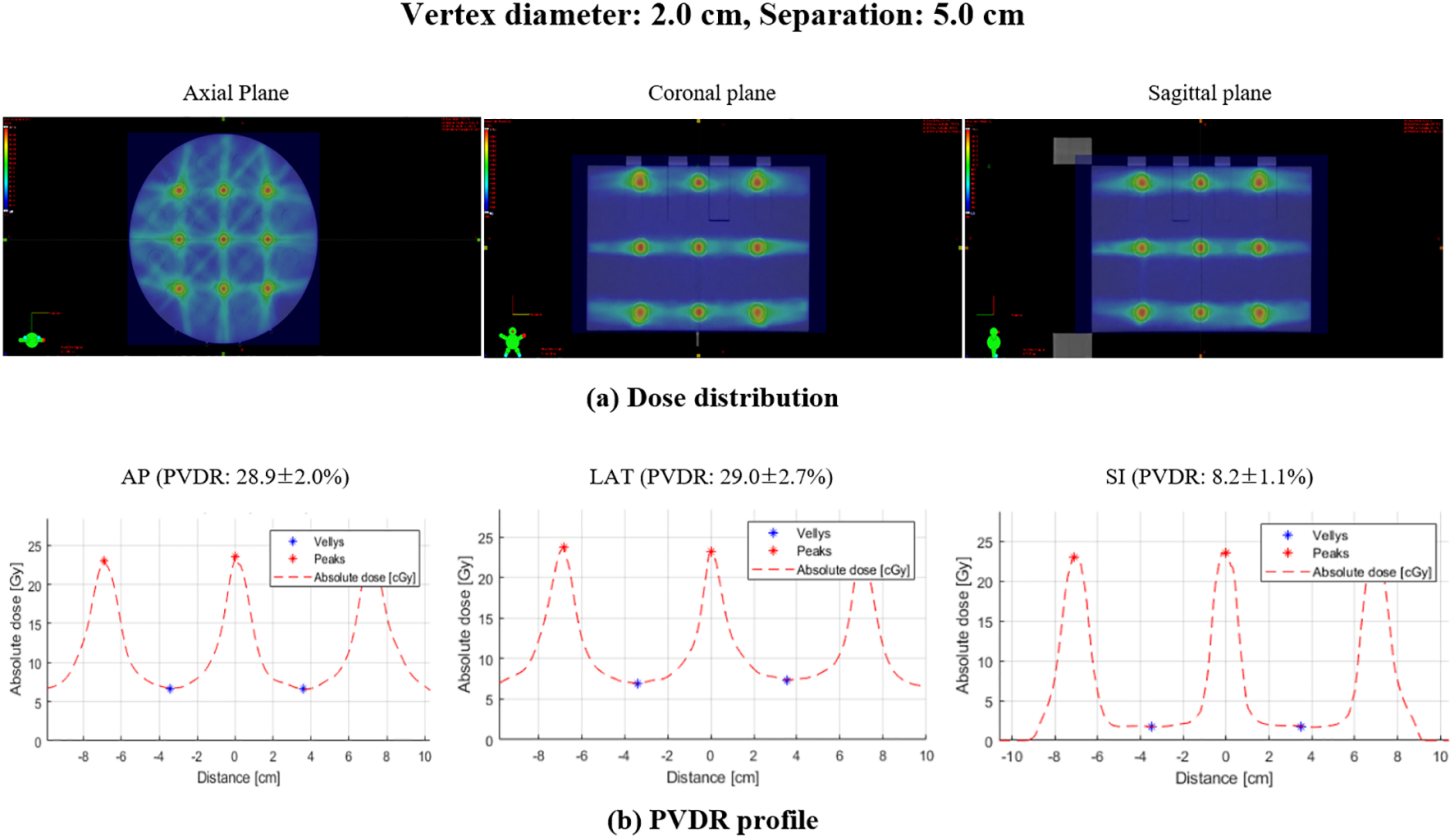
Dose distribution and PVDR profiles for lattice radiation therapy with 2.0 cm diameter and 5.0 cm separation: **(A)** dose distribution showing the spatial arrangement of high and low dose regions, **(B)** PVDR profile demonstrating the dose variation along the specified directions: AP, anterior-posterior; LAT, lateral; SI, superior-inferior.

The PVDR for LRT plans was evaluated based on varying vertex diameters (0.5 cm to 2.0 cm) and separations (1.0 cm to 5.0 cm) along the AP, LR, and SI directions. **Figure 4** illustrates the dose distribution and PVDR profiles deliverable with the TrueBeam equipped with MLCs, for a 2.0 cm vertex diameter and 5.0 cm separation.

**Figure 5:**
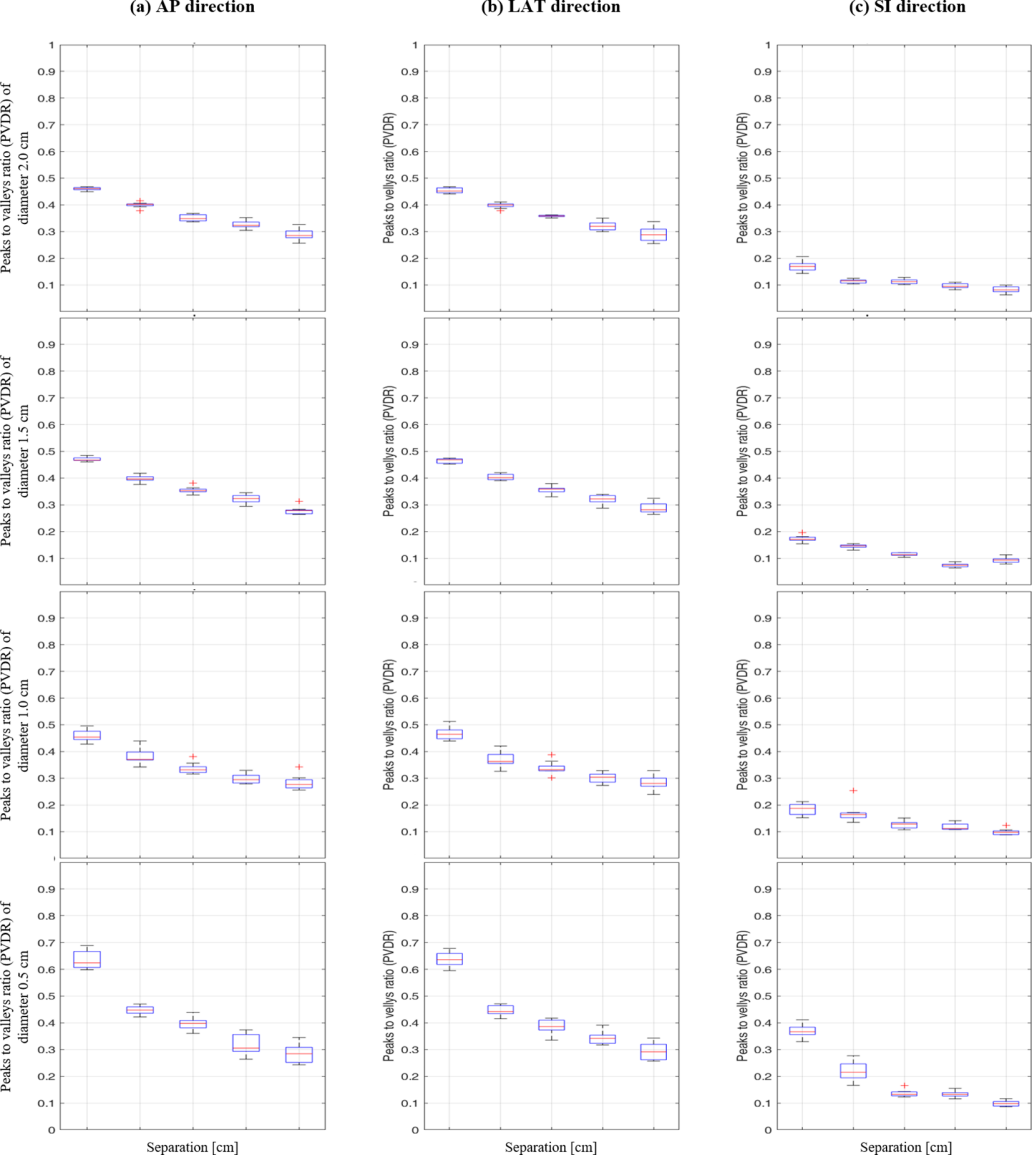
Box plot of peak-to-valley dose ratio (PVDR) results at different vertex diameters (0.5-2.0 cm) and separations (1.0-5.0 cm) for: **(A)** anterior-posterior, **(B)** lateral, and **(C)** superior-inferior directions.

As illustrated in **Figure 5**, the box plot analysis of PVDR data revealed consistent trends across all vertex diameters and directions, clearly showing the variations for different intervals and directions. As the separation between vertices increased, the PVDR consistently decreased in all directions (AP, LR, and SI). This trend was most pronounced in the SI direction, which showed the lowest PVDR values overall.

The impact of vertex diameter on PVDR was systematically analyzed, showing comprehensive PVDR values for different diameter and separation combinations (**Table 1**). For smaller separations (1.0 cm), increasing the vertex diameter from 0.5 cm to 2.0 cm resulted in a significant decrease in PVDR, particularly in the AP and LR directions. However, this effect became less pronounced at larger separations.

**Table 1:** Impact of diameter and separation of vertices on peak-valley dose ratio (PVDR) in lattice radiation therapy.

Peak-valley dose ratio (%)						
Diameter (cm)	Direction	Separation (cm)				
		1	2	3	4	5
0.5	AP	63.4 ± 3.4	44.8 ± 1.7	39.6 ± 2.5	32.0 ± 3.8	28.3 ± 3.4
	LAT	63.7 ± 2.9	44.6 ± 1.9	38.7 ± 2.7	34.2 ± 2.5	29.1 ± 3.2
	SI	37.0 ± 2.6	22.0 ± 3.5	13.7 ± 1.3	13.4 ± 1.1	9.9 ± 1.0
1.0	AP	45.8 ± 2.2	38.2 ± 2.8	33.6 ± 2.1	29.8 ± 1.7	28.4 ± 2.7
	LAT	46.8 ± 2.3	37.0 ± 2.7	33.7 ± 2.5	30.1 ± 1.8	28.4 ± 2.8
	SI	18.3 ± 2.2	16.9 ± 3.4	12.5 ± 1.4	11.8 ± 1.2	9.8 ± 1.1
1.5	AP	47.0 ± 0.7	39.7 ± 1.2	35.5 ± 1.2	32.1 ± 1.7	27.8 ± 1.5
	LAT	46.5 ± 0.9	40.3 ± 1.1	35.5 ± 1.5	32.1 ± 1.7	28.7 ± 1.9
	SI	17.3 ± 1.2	14.5 ± 0.8	11.4 ± 0.6	7.3 ± 0.7	9.3 ± 1.1
2.0	AP	46.0 ± 0.6	40.0 ± 1.0	35.1 ± 1.2	32.5 ± 1.5	28.9 ± 2.0
	LAT	45.4 ± 1.0	39.8 ± 1.0	35.8 ± 0.4	32.1 ± 1.7	29.0 ± 2.7
	SI	17.0 ± 1.8	11.4 ± 0.7	11.3 ± 0.9	9.7 ± 1.0	8.2 ± 1.1

Interestingly, the AP and LR directions showed similar PVDR values and trends across all conditions, while the SI direction consistently exhibited lower PVDR values. This directional dependence was particularly evident for the smallest vertex diameter (0.5 cm), where the difference in PVDR between AP/LR and SI directions was most pronounced.

These findings provide crucial insights for optimizing LRT treatment plans, highlighting the importance of considering both vertex size and separation, as well as directional effects, in achieving desired PVDR distributions

### 3.2 Normal tissue dose and volume analysis by diameter and separation

The distribution of dose in normal tissues was evaluated using LRT with varying diameters and separation conditions, providing detailed analysis of mean dose and intersecting volume across different configurations (**Table 2**). The results for the Eval_Normal_ROI, including mean dose and intersecting volume, were analyzed.

**Table 2:** Mean Dose and Intersecting Volume for Different Diameters and Separations.

Diameter (cm)	Evaluation items	Separation (cm)				
		1	2	3	4	5
0.5	Mean dose (cGy)	70.9	93.3	123.3	155.2	173.8
	Intersecting volume (cm^3^)	5.8	2.9	2.1	1.4	1.3
1	Mean dose (cGy)	106.4	144.9	182.2	202.9	220.1
	Intersecting volume (cm^3^)	62.5	58.4	58.3	58.1	14.4
1.5	Mean dose (cGy)	157.5	187.1	228	238.7	298.6
	Intersecting volume (cm^3^)	99.1	95.2	91.2	58.3	25.7
2	Mean dose (cGy)	223.2	258.6	298	337.5	355.6
	Intersecting volume (cm^3^)	375.2	371.4	325.1	181.6	140.2

#### 3.2.1 Variation of mean dose with diameter

The mean dose increased with the diameter. For a diameter of 0.5 cm, the mean dose was 70.9 cGy at a separation of 1.0 cm and increased to 173.8 cGy at a separation of 5.0 cm. For a diameter of 1.0 cm, the mean dose was 106.4 cGy at a separation of 1.0 cm and rose to 220.1 cGy at a separation of 5 cm. Similarly, for diameters of 1.5 cm and 2.0 cm, the mean dose increased with the diameter. Specifically, at a diameter of 2.0 cm, the mean dose started at 223.2 cGy at a separation of 1 cm and increased to 355.6 cGy at a separation of 5 cm. This indicates that larger diameters result in more doses delivered to normal tissues.

#### 3.2.2 Variation of intersecting volume with diameter

The intersecting volume varied with diameter and separation. The intersecting volume was defined as the volume where the Eval_Normal_ROI overlaps with the region receiving 50% or more of the prescription dose (10.0 Gy of the 20.0 Gy prescription dose). For a diameter of 0.5 cm, the intersecting volume was 5.8 cm³ at a separation of 1 cm and decreased to 1.3 cm³ at a separation of 5 cm. For a diameter of 1 cm, the intersecting volume was 62.5 cm³ at a separation of 1 cm and reduced to 14.4 cm³ at a separation of 5 cm. With a diameter of 1.5 cm, the intersecting volume started at 99.1 cm³ at a separation of 1 cm and decreased to 25.7 cm³ as the separation increased. For a diameter of 2 cm, the intersecting volume was highest at 375.2 cm³ with a separation of 1 cm and decreased to 140.2 cm³ at a separation of 5 cm. These results show that as the separation increases, the intersecting volume with normal tissues decreases. However, as the diameter increases, the intersecting volume increases.

### 3.3 Impact of diameter and separation on MU and MCS

The treatment plan was created for implementing LRT using the medical linear accelerator with coplanar arcs at four different collimator angles (0.0°, 45.0°, 90.0°, or 315.0°). The total MU was calculated based on diameter and separation, and the contribution of each collimator angle to the overall MU was analyzed.

**Figure 6:**
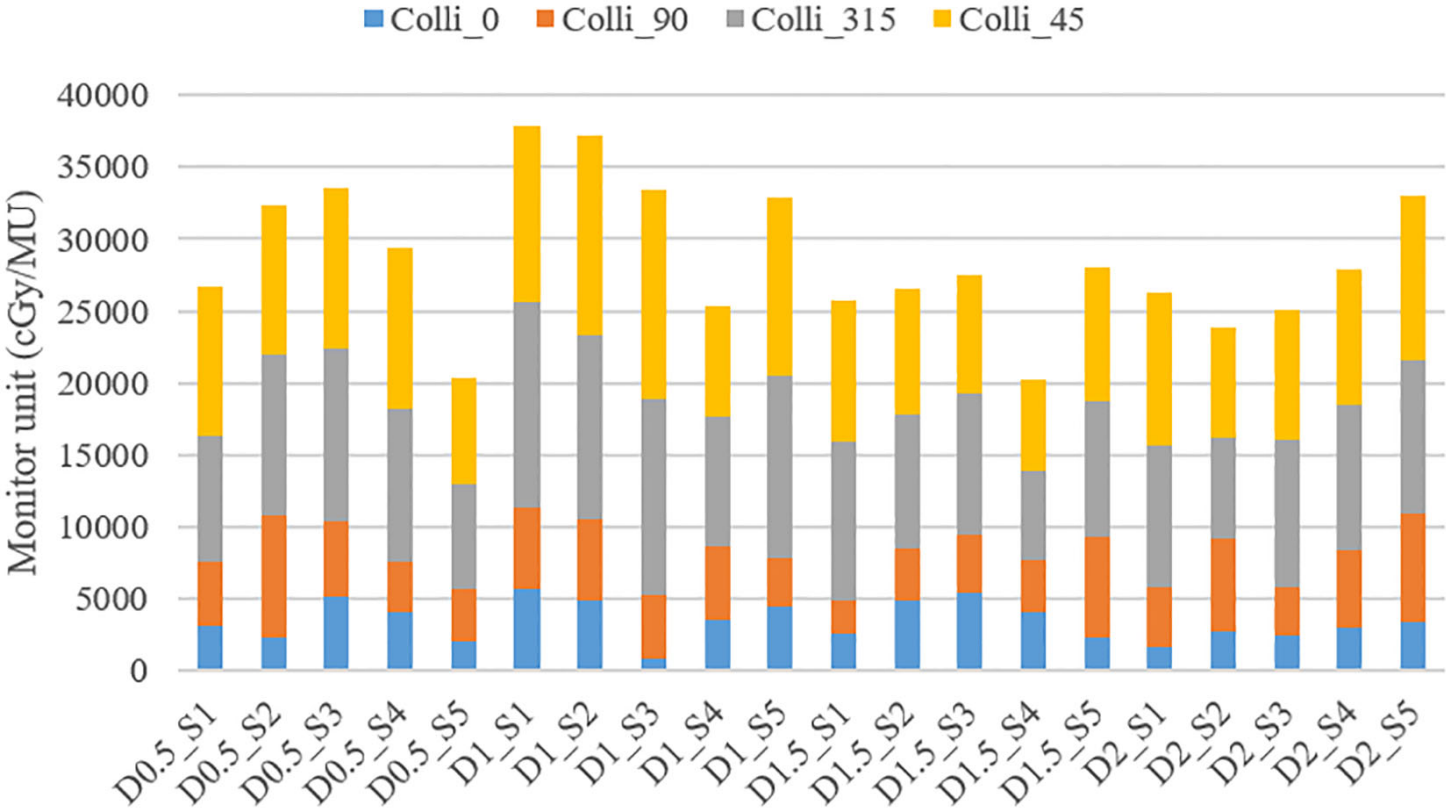
Variation in Total MU and MU contributions from different collimator angles (0.0°, 45.0°, 90.0°, or 315.0°) according to vertex parameters (diameter and separation).

As shown in **Figure 6**, the changes in total MU values can be observed for various combinations of diameter and separation. The total MU values varied between 20176.6 MU and 37908.9 MU, though no distinct pattern was observed in relation to changes in diameter and separation.

The detailed analysis of MU contributions from different collimator angles revealed that collimator angles of 315.0° and 45.0°consistently showed the highest MU contributions (**Table 3**). For instance, in the treatment plan with 1.0 cm diameter and 3.0 cm separation (D1_S3 plan), the collimator at 315.0°contributed 13596.5 MU (40.74% of the total), while the collimator at 45.0°contributed 14534.6 MU (43.56% of the total), together accounting for 84.29% of the overall MU. This trend was similarly observed across other diameter and separation combinations. In contrast, collimator angles of 0° and 90° generally demonstrated relatively lower MU contributions. In the same D1_S3 plan, the collimator at 0°contributed 891.8 MU (2.67% of the total), and the collimator at 90°contributed 4349.6 MU (13.03% of the total).

**Table 3:** Variation in total MU and MCS with changes in diameter and separation.

	Total MU	Collimator_0		Collimator_90		Collimator_315		Collimator_45		MCS
		MU	%	MU	%	MU	%	MU	%	
D0.5_S1	26673.9	3165.2	11.87	4389.7	16.46	8801.8	33	10317.2	38.68	0.02
D0.5_S2	32383.4	2290	7.07	8568.2	26.46	11143.3	34.41	10381.9	32.06	0.06
D0.5_S3	33533.2	5147.1	15.35	5277.7	15.74	12009.6	35.81	11098.8	33.1	0.09
D0.5_S4	29375.7	4002.2	13.62	3515.3	11.97	10632.7	38.21	11225.5	38.21	0.11
D0.5_S5	20339.6	1983.9	9.75	3730.5	18.34	7251.8	35.65	7373.4	36.25	0.17
D1_S1	37908.9	5671.7	14.96	5643	14.89	14309.6	37.75	12284.6	32.41	0.20
D1_S2	37244.7	4866.7	13.07	5594.8	15.02	12793.3	34.35	13989.9	37.56	0.09
D1_S3	33372.5	891.8	2.67	4349.6	13.03	13596.5	40.74	14534.6	43.56	0.12
D1_S4	25381.1	3557.6	14.02	5126.1	20.2	8966.2	35.33	7731.2	30.46	0.17
D1_S5	32914.9	4473.6	13.59	3340	10.15	12677.3	38.52	12424	37.75	0.08
D1.5_S1	25721.7	2522.2	9.81	2328.1	9.05	11087.2	43.1	9784.2	38.04	0.14
D1.5_S2	26582.9	4859.3	18.28	3662.4	13.78	9238.2	34.75	8823	33.19	0.09
D1.5_S3	27550.6	5422.3	19.68	4040.9	14.67	9877.1	35.85	8210.3	29.8	0.09
D1.5_S4	20176.6	4018.5	19.92	3736.5	18.52	6178.1	30.62	6243.5	30.94	0.08
D1.5_S5	28060.4	2302.7	8.21	7017.7	25.01	9489.4	33.82	9250.6	32.97	0.19
D2_S1	26234.6	1686	6.43	4070.4	15.52	9923.6	37.83	10554.6	40.23	0.10
D2_S2	23912.5	2692.3	11.26	6505	27.2	7020.3	29.36	7694.9	32.18	0.16
D2_S3	25084.9	2381.9	9.5	3387.6	13.5	10245.3	40.84	9070.1	36.16	0.11
D2_S4	27883.1	3035.2	10.89	5288.6	18.97	10188.6	36.54	9370.7	33.61	0.11
D2_S5	33025.8	3451.4	10.45	7514.1	22.75	10622.1	32.16	11438.2	34.63	0.11

These results suggest that collimator angles of 315.0° and 45.0° play a dominant role in the distribution of radiation dose in SFRT implementation. Although the effect of changes in diameter and separation on the total MU did not exhibit a consistent pattern, the choice of collimator angles was found to have a decisive impact on the distribution of the overall MU.

MCS analysis revealed that, for the smallest vertex diameter (0.5 cm), MCS values increased from 0.02 to 0.17 as separation distance increased, indicating that closer vertex spacing resulted in higher plan complexity. In contrast, for larger vertex diameters (1.0-2.0 cm), no distinct trend was observed regardless of separation distance.

## 4 Discussion

This study evaluated the feasibility of implementing LRT using a medical linear accelerator equipped with the Millennium 120 MLC, focusing specifically on a systematic investigation of 3×3×3 vertex configurations. By maintaining a fixed lattice structure of 27 vertices while varying vertex diameter and separation parameters, we aimed to establish fundamental relationships between these geometric parameters and achievable PVDR values. The results provide crucial insights into the potential clinical application of LRT, particularly in predicting achievable PVDR based on vertex configurations, which had not been previously investigated for this specific delivery system.

The PVDR analysis, a key focus of this study, offers valuable insights into the achievable dose distributions using this specific equipment for LRT implementation. This quantitative assessment of PVDR under various conditions of vertex diameter and separation is crucial for treatment planning, allowing clinicians to estimate PVDR outcomes before actual plan creation.

Our results can be compared with previous studies using similar vertex parameters. When considering vertex diameter of 1.5 cm, Gaudreault et al. achieved D90/D10 ratio of 35.5 ± 1.5% with center-to-center separation of 3.5 cm, which corresponds to our edge-to-edge separation of 2.0 cm showing PVDR values of 39.7 ± 1.2% and 40.3 ± 1.1% in AP and LR directions respectively (23). Ertan et al. also demonstrated similar results with the same vertex size and separation, reporting PVDR values of 33.7 ± 2.5% for CyberKnife and 37.0 ± 2.7% for VMAT delivery (24).

Our study uniquely contributes to PVDR estimation by systematically evaluating various separation distances and demonstrating directional dependencies. Notably, we found significantly lower PVDR values in the SI direction (14.5 ± 0.8% at 2.0 cm separation) compared to AP and LR directions. Additionally, our results show that increasing separation consistently leads to decreased PVDR values in all directions. Several studies including Ahmed et al. and Wu et al. suggested that clinically effective PVDR values should be maintained between 20% and 30% (12, 14). Our results align with this recommended range, particularly when using larger separation distances. For instance, with 5.0 cm edge-to-edge separation, we achieved PVDR values of 27.8 ± 1.5% and 28.7 ± 1.9% in AP and LR directions respectively. Notably, the PVDR values below 30% in all directions under the 5.0 cm separation condition are particularly encouraging. This suggests the potential to create a distinct contrast between high-dose (vertices) and low-dose regions within the tumor, maximizing the biological benefits of LRT. Such heterogeneous dose distribution can promote reoxygenation of hypoxic tumor regions and stimulate immune responses, potentially enhancing therapeutic efficacy.

Our analysis revealed important directional dependencies in dose distribution, which is critical information for LRT planning. The lower PVDR values observed in the SI direction compared to AP and LR directions indicate a steeper dose gradient in this direction. This phenomenon occurs due to the rotation of the gantry during treatment delivery. The vertices in the AP and LR directions lie on the same plane, resulting in uniform dose distribution across all vertices in this plane. In contrast, the SI direction, which follows the gantry’s rotation axis, experiences independent dose distribution for each vertex. This leads to relatively lower PVDR values in the SI direction, emphasizing the need for careful consideration of dose distribution in this direction during treatment planning.

The study also assessed the impact on normal tissue, another critical aspect of LRT feasibility. Using publicly available CT images from The Cancer Imaging Archive (TCIA) database (Colorectal Liver Metastases collection), we conducted a comparative analysis between LRT and conventional VMAT plans for a liver case with voluminous cancer: The total liver volume including PTV was 1741.2cc, and our target PTV volume was 394.4cc. The mean dose to normal liver tissue was 173.8 cGy and 834.1 cGy for LRT and VMAT, respectively. Moreover, the volume of normal liver tissue receiving high doses (>10 Gy) was substantially reduced in LRT compared to VMAT, with volumes of 3.3 cm³ and 465.4 cm³, respectively. This suggests that LRT has the potential to maintain normal tissue protection while allowing for adjustment of the balance between tumor control and normal tissue sparing.

**Figure 7:**
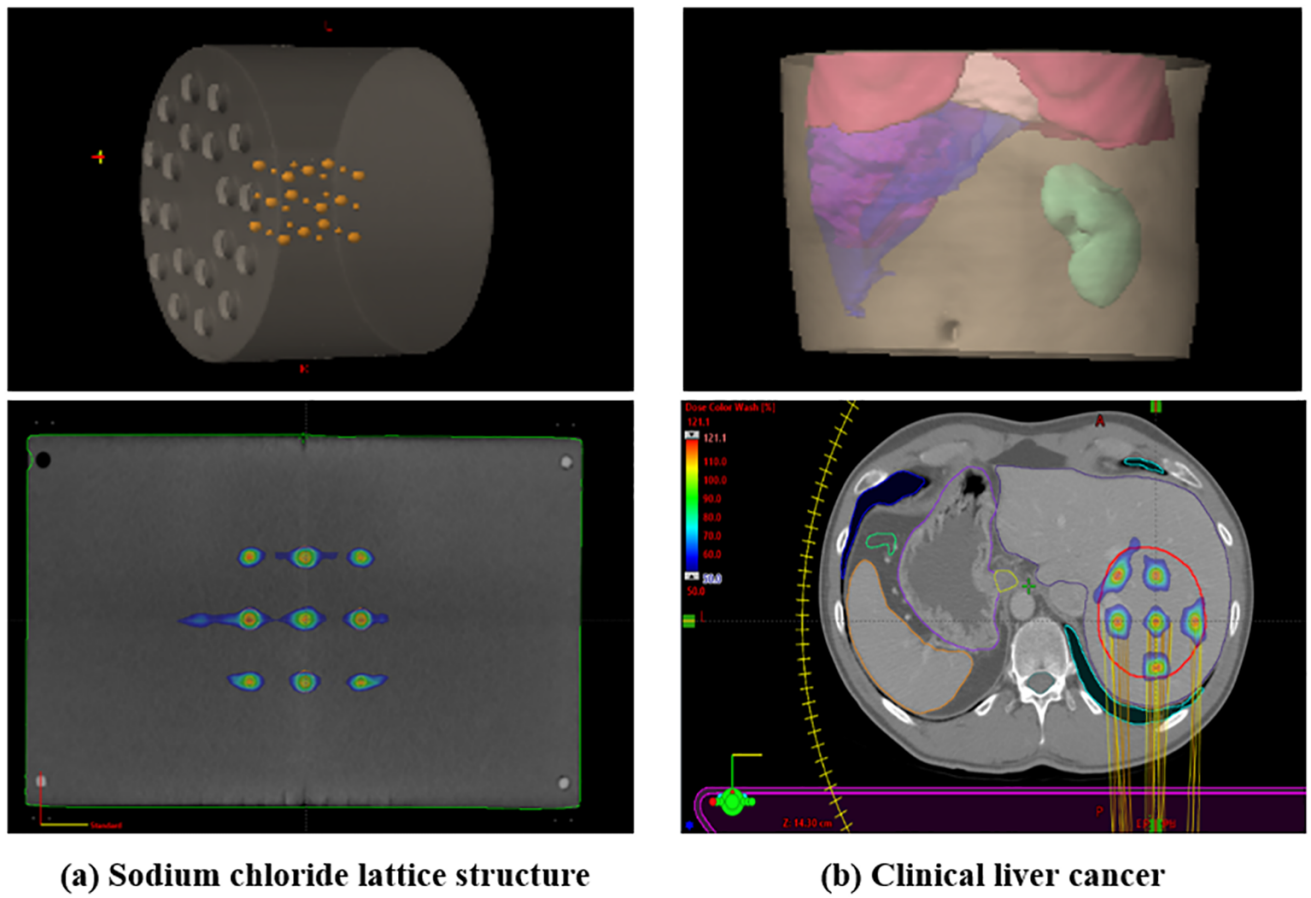
Demonstration of LRT application in **(A)** sodium chloride lattice structure with alternating vertex sizes (1.0 cm and 0.5 cm diameters with 3.0 cm center-to-center spacing) and **(B)** clinical liver cancer case. The inset images show 3D reconstructed views of each structure.

The total MU analysis, which showed high contributions from collimator angles of 315.0° and 45°, provides important information for the technical implementation of LRT. These angles appear to be particularly effective in generating the spatial fractionation pattern of LRT, which could serve as a valuable guideline for future LRT planning optimization. This pattern of MU contribution from collimator angles 315.0° and 45.0° was consistently observed across different geometric configurations, including a sodium chloride lattice structure (1.0 cm and 0.5 cm diameters with 3.0 cm center-to-center spacing) with alternating vertex sizes and a clinical liver cancer case, suggesting their importance in achieving desired SFRT dose distributions. As shown in **Figure 7**, we implemented these different geometric configurations, and **Table 4** demonstrates that both cases maintained similar patterns of MU contribution, with collimator angles 315.0° and 45.0°contributing 60.49% (33.44% and 27.05%) and 61.85% (27.78% and 33.07%) of the total MU for the sodium chloride structure and liver cancer case, respectively.

**Table 4:** Comparison of MU contributions from different collimator angles in sodium chloride lattice structure and clinical liver cancer cases.

	Total MU	Collimator_0		Collimator_90		Collimator_315		Collimator_45	
		MU	%	MU	%	MU	%	MU	%
Sodium chloride lattice structure	37705.9	8785	23.3	6115.3	16.22	12609.8	33.44	10195.8	27.05
Clinical liver cancer	19106.1	3353.3	17.55	4125.7	21.59	5307.8	27.78	6319.3	33.07

The MCS analysis provided additional insights into the relationship between geometric parameters and plan complexity. The analysis revealed that treatment plans showed higher complexity with closer vertex spacing in smaller diameter cases (0.5 cm), suggesting that careful consideration of separation distance is crucial for managing plan complexity when using smaller vertices. Conversely, larger diameters (1.0-2.0 cm) showed no clear trend with varying separation distances. When compared to McNiven et al.’s IMRT complexity study (22), which reported site-specific MCS values ranging from 0.909 for breast to 0.165 for head and neck treatments (with intermediate values of 0.823 for rectum, 0.739 for prostate, 0.580 for prostate bed, and 0.645 for lung), our study found MCS values ranging from 0.02 to 0.2. This reflects the inherently complex nature of SFRT dose distribution, which requires steep dose gradients between peaks and valleys to achieve the desired PVDR values of 20-30%.

A key strength of this study is its systematic evaluation of LRT feasibility across various vertex diameters and separation conditions, providing fundamental parameters for a clinical application of LRT. Furthermore, the implementation of LRT using the Millennium 120 MLC demonstrates the potential for utilizing existing radiation therapy equipment, suggesting the possibility of widespread adoption of LRT.

However, this study has several limitations. Being a phantom study without the use of actual patient data limits direct application to clinical settings. Moreover, the study did not directly assess the biological effects of LRT or organ-specific tolerances, which should be addressed in future research.

In conclusion, this study provides a valuable reference for predicting PVDR in LRT using standard linear accelerator equipment with MLC. By offering insights into the relationship between vertex configuration and resulting PVDR, this research enables clinicians to make informed decisions in LRT planning without the need for time-consuming trial-and-error approaches. Future research should focus on validation using real patient data, clinical studies on various tumor types and locations, and in-depth investigations into the biological effects of LRT. Through these efforts, LRT may open new horizons in cancer treatment, offering a more precise and potentially more effective approach to radiotherapy.

## 5 Conclusion

This study demonstrates the technical feasibility of implementing LRT using a medical linear accelerator equipped with the Millennium 120 Multi-Leaf Collimator. Key findings include achievable PVDR values under various vertex configurations, directional dependencies in dose distribution, and the relationship between vertex parameters and dose outcomes. These results provide a valuable reference for LRT treatment planning, potentially streamlining the optimization process. This study lays a foundation for future LRT development, potentially opening new avenues in cancer treatment by offering improved tumor control with normal tissue sparing.

## Data Availability

The original contributions presented in the study are included in the article/supplementary material. Further inquiries can be directed to the corresponding authors.
